# Towards the integration of ecophysiology with fisheries stock assessment for conservation policy and evaluating the status of the Mediterranean Sea

**DOI:** 10.1093/conphys/coac008

**Published:** 2022-03-11

**Authors:** F Falco, T Bottari, S Ragonese, S S Killen

**Affiliations:** Institute of Biological Resource and Marine Biotechnology (IRBIM), National Research Council (CNR), Section of Mazara del vallo, Via L. Vaccara, 61 91026, TP, Italy; Institute for Marine Biological Resources and Biotechnology (IRBIM), National Research Council (CNR), Section of Messina, 98122 Messina, Italy; Institute of Biological Resource and Marine Biotechnology (IRBIM), National Research Council (CNR), Section of Mazara del vallo, Via L. Vaccara, 61 91026, TP, Italy; Institute of Biodiversity, Animal Health and Comparative Medicine, University of Glasgow, Glasgow, G12 8QQ, UK

## Abstract

Recent European Union (EU) regulations have been introduced to discourage the capture of undersized specimens with the aim of reducing the bycatch mortality imposed by commercial fisheries. We argue that we still lack accurate data regarding basic information required to properly implement these regulations for most Mediterranean ecosystems, including the true mortality imposed by fisheries, escape rates from fishing gears and the capability of specimens to survive following discard. We suggest that additional reliance on physiological biomarkers could assist in all aspects of the data collection required to support implementation of the EU discard ban (aka landing obligation), particularly in determining which species should receive special dispensation from this policy. Ideally, this new approach, here termed the ‘Fisheries Environmental and Physiological Stress Analysis’ (FEPSA), would become an important step for any fish stock assessment within the ecosystem approach to fisheries management and the recognition of Good Environmental Status, as established by the EU in the Marine Strategy Framework Directive (2008/56/EC). In particular, the main goal of FEPSA would be applying the study of physiological stressors to exploited stocks to estimate the so-called collateral fishing mortality, which includes the mortality experienced by fish that escape after interacting with fishing gears or that are discarded, with some degree of injury or physiological stress. The approach outlined here, which is described for bottom trawls but adaptable to any other type of fishing gear, is not a trivial undertaking but is a requirement for collecting the data required by recent EU fisheries policies. While we agree that the threats to marine biodiversity posed by fishing and associated discard practices require strong policy interventions, we emphasize that the research programs needed to support such initiatives, including the landing obligation, should be given equal priority. This is particularly true for Mediterranean fisheries, which are at a complex intersection of jurisdictional boundaries, numerous additional ecosystem threats including widespread pollution, thermal variation and hypoxia, and are historically understudied as compared to fisheries and species in more northern climates.

## Lay Summary

Owing to the insufficient or inaccurate information regarding the overall effective fishing mortality caused by fishing effort, gear escape rate and discard practices—all information required by the recent European Union discard ban—we propose the development of an approach based on accurate physiological biomarkers for estimation of stress as an index of collateral mortality.

## Background

Overfishing and corresponding population declines are one the most serious threats to marine biodiversity. Yet, there is a range of potential indirect effects that fishing may exert on wild fish populations, aside from the direct mortality of fish that are removed from the population as part of the landed catch. Natural predators are known to produce non-consumptive effects on prey populations by the generation of physiological stress in prey, infliction of injuries and by affecting nutrient intake via trophic cascades or threat-sensitive foraging. In turn, these mechanisms can alter prey growth rate and reproductive output or produce indirect mortality. While these indirect effects of predation are appreciated in ecology and believed to affect entire populations and ecosystems via trait-mediated effects, a detailed understanding of the indirect effects of fishing—in which humans are the predators and fish are the prey—is still lacking. Without such knowledge, it is extremely difficult to predict how policies aiming to reduce the impactors of fishing will affect wild populations.

**Figure 1:**
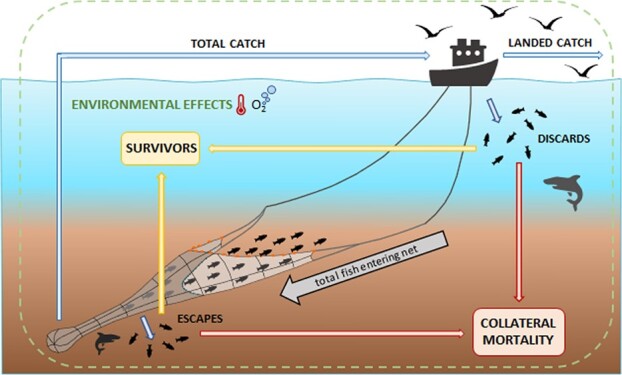
A schematic of the possible fates of fish involved in a large-scale fishing event. This illustration uses trawling as an example, but analogous classifications of fish experiencing the various fates could also be used for fish targeted by other gears including seines, longlines, pots or traps. During a trawl, fish swimming in front of the gear will experience some stress even if they avoid final capture. The portion of fish that enter the net will be subdivided into those that either escape (by avoiding the trawl or passing through the mesh after entering the net) or are brought aboard the fishing boat. Fish that are captured (brought on board) are either discarded or retained (landed). Fish that escape during the final stages of net hauling or just before the gear is placed on board (e.g. slipping; not represented), together with those fish that are discarded can either recover and survive or experience indirect/collateral fishing related mortality; the latter may occur directly from the physiological disturbance incurred during the capture process, or indirectly (e.g. from predation), due to behavioural impairments during recovery (Schmitz and Suttle, 2001; Barton and Schmitz, 2009). Environmental factors (indicated by the green dashed box), such as the prevailing water/air temperature or water oxygen availability, will have an overriding effect on fish physiology, behaviour and therefore the various responses (Sopinka *et al.,* 2015).

Recently, the European Union (EU) has introduced regulations to minimize fishing-associated mortality, especially that stemming from commercial fishery discards. The primary regulation is the discard ban, a.k.a., landing obligation (EU Delegate Regulation No. 2015/2439; STECF, 2016), which is the obligation of retaining and landing the undersized specimens of some species for which a minimum conservation reference size (MCRS) has been established. This is a step towards addressing the major conservation issue of bycatch by commercial fisheries, allowing definitive quantification of this source of mortality in relation to overall fishing mortality. In turn, this will facilitate more accurate estimation of whether mortality among commercially exploited species is at or below levels which can produce the maximum sustainable yield. In most Mediterranean jurisdictions, the fishing mortality of demersal species is usually estimated via the size structure of commercial catches and then fine-tuned with data from scientific bottom-trawl surveys (Spedicato *et al.,* 2019). However, the catch resulting from a given fishing effort may not be the most appropriate index of the overall fishing mortality (ICES, 2011; Kaiser *et al*., 2002; Broadhurst *et al.,* 2006), because many additional fish may die after escaping fishing gears or after being discarded with injury or physiological stress, experiencing the so-called collateral (hidden or unaccounted) fishing mortality. Marine organisms can experience physiological effects throughout the fishing process, ranging from stress induced during the initial approach of an active fishing gear (e.g. due to boat noise) to the gear confinement and potential escape (e.g. in pot traps, seines, trawls) and potential discard after capture. Collateral mortality is not easy to estimate because it varies among species, populations, fishing techniques, gear types and environmental variables. All these factors may interact to determine the physiological stress and physical injury that fish experience while interacting with gears that influence their potential for recovery (Fig. 1).

**Figure 2:**
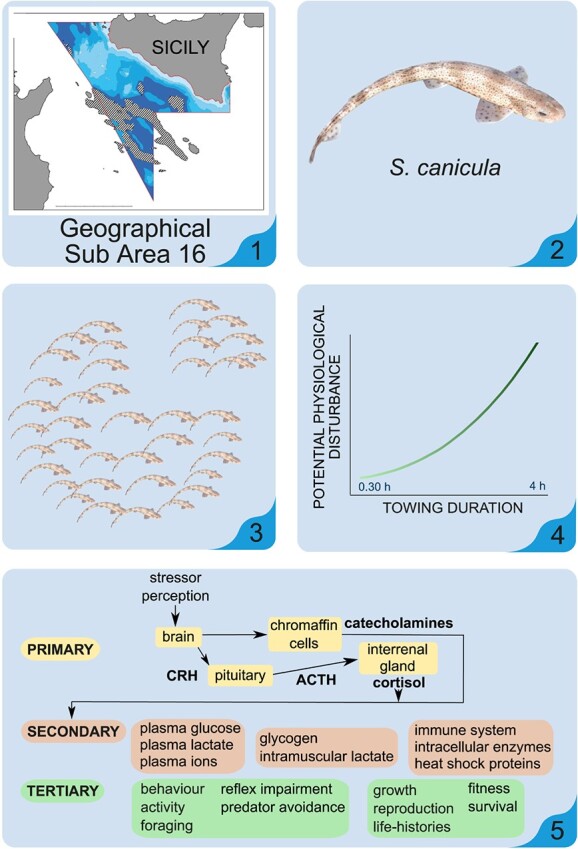
Steps involved in the exploratory/preliminary phase of FEPSA. The approach described here is specific to experimental trawling but could be adapted to other fishing gears. The overall goal of this phase is to assess biomarkers of physiological disturbance and to calibrate these biomarkers for behavioural disturbance, effects on growth and fitness, or survival. (i) Identify a geographical sub area of interest (e.g. GSA16 in the specific case), ideally where the environmental conditions are relatively homogenous, allowing the assumption of similar levels or natural or ‘baseline’ stress experienced by fish within the region. (ii) For initial evaluation, select a species anticipated to be robust to onboard manipulation (e.g. *Scyliorhinus canicula*) to more easily pinpoint the effects of various environmental factors (e.g. temperature) and fishing practices, without having these effects be overwhelmed by stressors encountered during handling and sampling.(iii) Using a representative sample of the population, subject experimental groups of fish to (iv) different experimental fishing procedures representing different fishing practices or potential levels of physiological (e.g. variation in trawl towing duration or trawl frequency). (v) Evaluate various biomarkers by examining relationships between physiological measures (indicative of activation of the primary and secondary stress responses) and tertiary responses. Effects on behaviour, growth, fitness and survival are challenging to evaluate but may be performed by monitoring of fish in enclosures or post-release using various tagging techniques (including acoustic telemetry).

**Figure 3:**
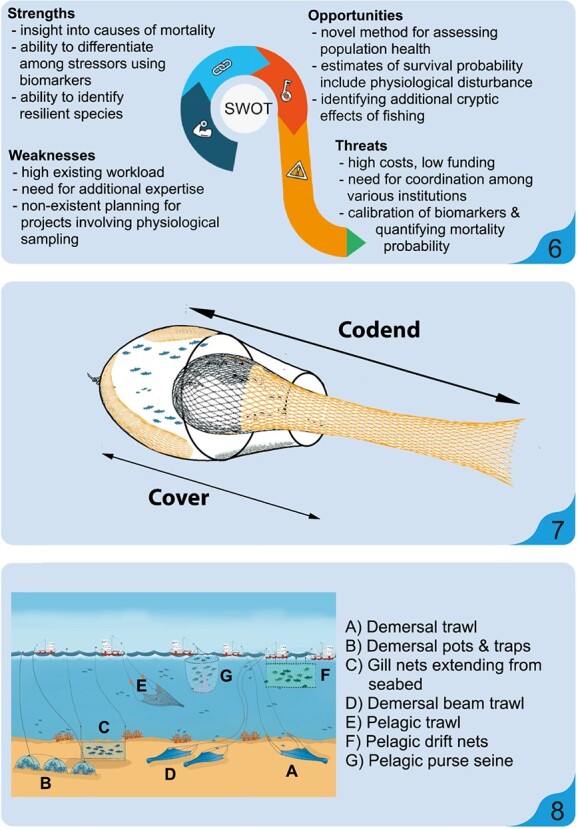
Based on the results of the exploratory/preliminary phase of FESPA described in Fig. 2, the monitoring/survey phase may proceed with the following steps: (vi) Due to the cost and labour requirements, a full sampling design should be conducted alongside a risk-assessment of the potential benefits, costs and pitfalls that may be experienced to obtain reliable collateral mortality (Fcol) estimates. (vii) Sampling of fish at various stages throughout the fishing process to evaluate the numbers of fish experiencing each phase (e.g. escape), the degree of physiological disturbance incurred and estimation of mortality probability using suitable biomarkers (identified during the exploratory/preliminary phase). This could include sampling of individuals escaping from a trawl codend (pictured in figure, with escapees retained using a trawl cover), but individuals could be sampled at any phase of the capture sequence and sampled for biomarker analysis. (viii) If appropriate, design and implement analogous trials to estimate Fcol related to other fishing gears. Ultimately, all previous steps in the exploratory/preliminary phase and the monitoring/survey phase can be repeated and refined for other species frequently captured by commercial fisheries.

While the discard ban is an important advance towards combating the worldwide problem of fisheries bycatch and discards, there remain several concerns regarding the implementation of this policy (Sardà *et al.,* 2015). For example, a species or stock can be granted special dispensation from the landing obligation if it has a high likelihood of survival following escape or discard from fisheries or has protected status. Unfortunately, the discard ban regulation does not specify the minimum survival requirements to achieve this dispensation, and so accurately estimating collateral fishing mortality has become a research priority. The goal is to determine not only which species are most and least vulnerable to sources of collateral mortality, but also the potential ranges of mortality in the first place, among species and in response to different fishing practices and environmental conditions. Currently, there is very little quantitative data on post-escape and post-discard survival for most Mediterranean demersal fish species and so there is no basis on which species to issue discard ban exceptions (or avoid a future inclusion of a new species in the discard ban) in this region. Likewise, although the proportion of fish retained by trawls and other gears can vary widely (Sánchez *et al.,* 2004), with many fish escaping capture at various points along the capture sequence (Suuronen, 2005; Hollins *et al.,* 2018), we have little knowledge of the extent of stress and mortality experienced by fish during escape after interacting with commercial fishing gears, especially for Mediterranean fisheries.

Aside from use in stock assessments, accurately quantifying total fishing mortality by including collateral mortality would be beneficial for understanding the strength of selection generated by fishing and the potential for fisheries-induced evolution (Enberg *et al.,* 2009; Heino *et al.,* 2013; Hollins *et al.,* 2018; Jørgensen *et al.,* 2007). Furthermore, as established by the EU Marine Strategy Framework Directive (2008/56/EC; Directive, 2008), the criteria for achieving officially recognized Good Environmental Status (GES) by the EU do not only include maintaining productive and economically viable fisheries, but also encompasses the welfare of the other marine ecosystem components, such as biodiversity and seascape conservation. An important component of this evaluation is an index derived from the assessment of 11 environmental descriptors (defined in regulation 477/2010/EU, September 2010 EC). These descriptors cover a wide range of parameters (Fiorentino *et al.,* 2013) both biotic (growth, mortality, reproduction, etc.) (Lassen *et al*., 2013) and abiotic (environmental features and socio-economic aspects). The evaluation of GES within this framework is a formidable challenge, especially throughout the large marine ecosystem of the Mediterranean Sea (Raicevich *et al*., 2017), where there are numerous fisheries for a wide variety of exploited species, research initiatives that are often uncoordinated and complicated jurisdictional boundaries with potentially conflicting interests (e.g. European and non-European countries).

**Figure 4:**
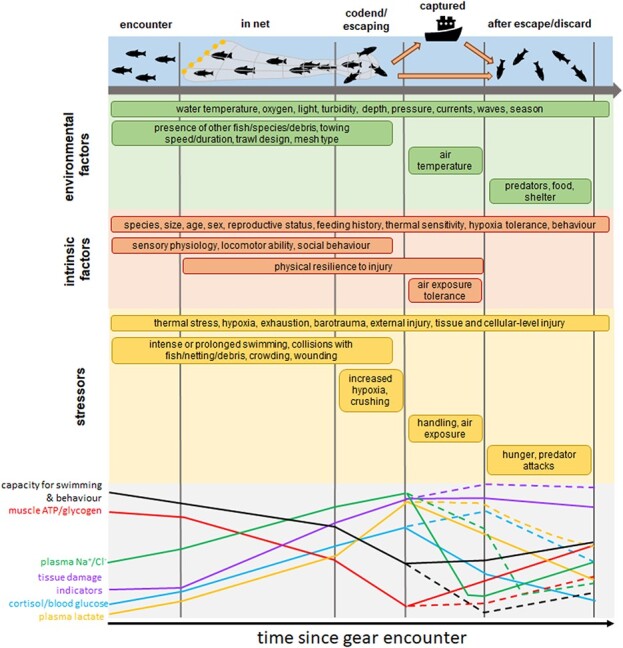
Depiction of the various stages a fish will encounter during the process of being captured by a trawl and experiencing escape or discard. This illustration uses trawling as an example, but many of the stressors and physiological responses could also occur for fish targeted by other gears including seines, longlines, pots or traps. The figure is adapted from Suuronen (2005) and Gilman *et al.* (2013); here specifically isolating extrinsic and intrinsic factors that could modulate the degree of stress experienced during each stage and the associated response of specific physiological and behavioural biomarkers of stress (grey box, bottom). Colours of different lines within the grey box show the potential theoretical response of each biomarker throughout the escape/discard experience. For simplicity, changes in the relative magnitude of each factor are shown as being linear, but this may not necessarily be the case. Solid coloured lines show trajectories for fish that interact with or enter the trawl, and then escape before being brought aboard a boat. Dashed lines indicate trajectories for fish that are retained within the trawl and are brought aboard, before being discarded. In general, fish that are brought on board can be expected to show a greater degree of physiological and behavioural disturbance and a longer absolute time until recovery. The initial rise in plasma Na+/Cl- concentration is due to haemo-concentration, as water enters the white muscle during physical activity due to increasing muscular lactate concentrations. Absolute plasma Na^+^/Cl^−^ eventually drops, leading to impaired physiological function, as ions are lost via the gills and water gradients are re-established. Injuries or stress may also increase susceptibility to disease or parasites, but this is not illustrated for simplicity.

In this Perspective, we argue that an increased use of physiological research methods and biomarkers could be key for more accurately estimating total ‘effective’ fishing mortality (F_o_) in Mediterranean ecosystems. This approach, here termed the ‘Fisheries Environmental and Physiological Stress Analysis’ (FEPSA; Figs 2 and 3), would apply the study of physiological stressors in exploited stocks to estimate the collateral mortality derived from fish escapees and discards (Benoît *et al.,* 2010; Davis, 2010). By analysing suitable physiological biomarkers in fish (Table 1 and Fig. 4), this approach would allow estimation of the collateral mortality to be added to the total fishing mortality. The application of physiological biomarkers to the assessment of potential collateral mortality would correspond to utility of physiology in marine conservation described by McKenzie *et al*. (2016) and the general framework and steps of FEPSA could be as follows in Figs 2 and 3.

The overall goals of FEPSA would be to (i) estimate the overall collateral mortality to be added to the current fishing mortality within a given fishery area, improving the relationship between effective fishing effort and overall fishing mortality (Alverson and Hughes, 1996; Chopin *et al.,* 1996; Harley *et al.,* 2000; Jean, 1963; McLoughlin *et al.,* 1991); (ii) give some evidence as to which species may be most likely to recover from stressors encountered during fishing and survive or to develop means by which to increase their chances of survival; (iii) highlight how other environmental stressors (including increased water temperature or hypoxia) may interact with fishing-associated stressors to influence collateral mortality; and (iv) allow more accurate scoring of GES, as per EU directives. The over-arching approach of FEPSA would be to use physiological biomarkers, from fish exposed to gear escape or discard scenarios, to estimate the collateral mortality caused by these practices and allow the optimal level of fishing effort to achieve maximum sustainable yield to be more accurately estimated (Gulland and Boerema, 1973; Penn *et al*., 1995; Froese *et al.,* 2016).

To estimate the overall mortality caused by escapes and discards, we essentially need to multiply the total number of fish that experience escape or discard by the physiologically informed probability of survival in each instance. This, in turn, requires several complementary research endeavours, which are outlined below. To be clear, this is not a trivial process, but more accurate estimates of mortality depend on information of this sort, as do the criteria for possible species dispensation from the landing obligation or avoid the inclusion of new species in the least list. Our description here is largely conceptual and outlines the research approach that is needed to adequately implement dispensations to the EU discard ban and in response to the urgent need but almost complete absence of research on the physiological responses of exploited Mediterranean fish species to interactions with fishing gears and discard.

**Table 1:** Synopsis of indicators/biomarkers and their capability for revealing information on stressors experienced by fish exposed to fishing procedures.

Biomarker	Stress indication	Sampling involved	Logistical challenges	Relative cost
**Primary response:** neuroendocrine responses to stressors and stimulation of the hypothalamic–pituitary–interrenal axis				
Catecholamine	Catecholamines are responsive to a variety of stressors (Reid *et al*., 1998; Pottinger, 2008); their measurement can provide information about the response to acute stressors at a fine temporal scale.	Typically measured in plasma^b^	Requires specialized equipment and personnel. Often not logistically possible to measure in the field because they are highly responsive to capture and handling.	Low
Cortisol	Cortisol responds more slowly than catecholamines to specific stressors, taking longer to elevate (minutes to hours) above pre-stressor levels.	Typically measured in plasma^b^	Can be quantified in laboratory or field settings. Requires specialized equipment and technicians. Can be responsive to capture and handling.	Medium
**Secondary response**: stress-related responses in plasma, tissues and organs				
Haematocrit	Increases due to splenic contraction to enhance blood O_2_ carrying capacity (McDonald and Milligan, 1992) and catecholamine activation of red blood cell (RBC) Na^+^–H^+^ exchangers, which tightly mediate and conserve optimal intracellular pH (Nikinmaa, 1992).	Measured in blood^b^	Can be quantified in laboratory or field settings. Requires specialized equipment and technicians.	Medium
Heat shock proteins (index of cellular stress)	Increases to maintain cellular homeostasis (Iwama *et al*., 2004) or to repair/catabolize proteins (Moseley, 1997). Sensitive to a range of stressors (e.g. rapid temperature changes, salinity challenges, handling; Palmisano *et al*., 2000; Donaldson *et al*., 2008)	If extracted from blood^b^; if other tissues are used^a^	Requires a specialized technician.	Medium
Intracellular enzymes (ALT-AST-LDH-CK)	Useful indicators that tissue damage has occurred (Morrissey *et al*., 2005; Butcher *et al*. 2011; Rapp *et al*., 2012), possibly indicative of severe or life-threatening trauma.	Samples taken from plasma (Wells *et al.,* 1986) or skin mucus (Piazzese *et al*., 2019)^b^	Requires a specialized technician for sample analysis.	Medium
Glucose	Increases in the blood following exposure to a stressor (Barton, 2002).	Measured by plasma or in whole blood (Wells and Pankhurst, 1999; Beecham et al., 2006; Stoot *et al.,* 2014)^b^	Can be measured in the field using properly calibrated portable metres or kits. Can change in response to many stressors so is non-specific and sensitive to capture and handling.	Low
Lactate	Rises during anaerobic metabolism, following hypoxia or bouts of intense physical activity (Wood *et al.,* 1983; Barton, 2002)	If measured in plasma^b^; for other tissues such as in the skeletal muscle^a^	Can be measured in the field using properly calibrated portable metres or kits.	Low
Osmolality and ionic concentration	Related to ions transfer at the gills, and subsequent changes in plasma osmolality (mainly Na^+^ and Cl^−^); good indicators of acute stress. Other ions as (K^+^, Ca^2+^, Mg^2+^) may also be affected. Plasma presence of intracellular ions may indicate severe or life-threatening trauma (Cliff and Thurman, 1984; Wells *et al.,* 1986)	Measured in plasma^b^	Specialized personnel required for sampling. Response and recovery can be protracted and confounded by haemoconcentration, so care must be taken during data interpretation.	Low
**Tertiary response**: stressor effects on whole-animal performance				
Reflex indicators (such as the ability to flip upright)	Neurological responses of fish to external stimuli or functions of the autonomic nervous system (Davis, 2010) Survival stress (Uhlmann *et al*., 2016).	Can be assessed individually (as present or absent) or as a composite of sub-responses to derive a score (Davis, 2010)^b^	Does not require any specialized equipment and provides an immediate (20 s) measure of fish vitality.	Low
Behaviour	Acoustic telemetry or accelerometry can reveal changes in fish behaviour following a stressor, including change sin spontaneous activity, foraging, or susceptibility to predation.	Requires surgery to attach or implant a data logger or transmitter^b^	May require long-term studies. Requires specialized equipment and personnel. Data analysis can be challenging and requires experienced personnel.	High
Growth and other life history traits (LHTs)	LHTs can be altered by chronic stress (e.g. reduced growth rate; Pankhurst and Van der Kraak, 1997). Can be indicative of population-level effects of stress.	Can be inferred by tagging and recapturing individuals or monitoring growth and reproduction in enclosures^b^; growth can also be estimated via analysis of otoliths^a^	May require long-term studies. Requires specialized personnel for data acquisition and expert researchers for data interpretation.	Medium–high
Reproductive timing, output and fecundity	Chronic stress can reduce the energy invested in reproduction. Can reveal sex-specific effects. Can be indicative of population-level effects of stress.	Can be monitored in enclosures^b^; can be estimated by measuring gonadosomatic index, gamete size/number.^a^	May require long-term studies. Requires specialized personnel for data acquisition and expert researchers for data interpretation.	Medium–high
Survival	The most extreme response to a stressor is death, whereby homeostasis cannot be maintained (Wood *et al.,* 1983)	Can be monitored in enclosures or using tracking (e.g. acoustic telemetry) or mark-recapture techniques^b^	May require long-term studies. Requires specialized equipment and expert personnel.	Medium, due to management cost

Biomarkers are sorted based on whether they pertain to the primary, secondary or tertiary stress responses. Cost is evaluated qualitatively as low, medium or high; however, cost may vary for the same biomarker according to context.

^a^Invasive procedure, i.e. involving the death of the individual.

^b^Non-invasive, i.e. not involving damage and/or additional stress to the individual (the preferable option advised by European commission).

## How many fish escape gears or are discarded?

First, there needs to be accurate estimation of how many fish experience escape or discard versus those that are retained and officially landed, an aim which is a general challenge for fisheries (Ragonese and Morara, 2012; FAO , 2019a,b). The number of discards in a fishery can be determined from observations and records of fish that have been returned after capture, but the estimation of escapes is much more complicated. For trawl fisheries, the traditional approach being to enclose the trawl net or coded with a cover that can retain fish that escape by passing through the mesh (Duzbastılar *et al*. 2010, 2017). Although there are many inherent difficulties and potential biases in this approach (Gilman *et al.,* 2013; Suuronen, 2005), these studies can provide valuable insight into the numbers of fish that escape and how this may vary in response to factors such as body size, species and fishing practices. Recent advances in underwater cameras and associated modelling frameworks will also refine estimates of the numbers of fish escaping from fishing gears (Robert *et al.,* 2020; Simon *et al.,* 2020) and have been used to estimate escapes from passive gears such as pots or traps (Folkins *et al*., 2021). In the case of purse seines, large numbers of fish may be intentionally released (or ‘slipped’) during the latter stages of the seine to avoid bycatch of unwanted species or size classes, and fish that are released in this manner can experience increased mortality (Marcalo *et al*., 2006; Tenningen *et al*., 2012). While the quantification of fish escapees from trawls has received considerable research attention over the past two decades, notably, almost all attempts to quantify fish escapees from trawls and other gears have been performed in relatively northern latitudes and species (Broadhurst *et al.,* 2006). As a result, there is little known about the factors that influence fish escapees from trawls in Mediterranean fisheries (Metin *et al*., 2005; Duzbastılar *et al*., 2010, 2017).

## What is the physiological condition of fish after escape or discard?

Next, we require an estimate of the physiological condition of Mediterranean fishes after they have experienced escape from trawls or have been discarded. As fish encounter and interact with a trawl and are brought on board a ship, they are subject to numerous physiological stressors (Fig. 4). The effects of these stressors are likely cumulative (Davis and Ottman, 2006), or sometimes delayed, with fish showing a greater degree of physiological disturbance the farther they are along the capture process (Hollins *et al.,* 2018). A range of physiological studies are required to quantify the extent of the physiological disturbance experienced by individuals at each stage of this process and, ideally, how these responses are modulated by environmental factors (Killen *et al.,* 2013). Isolating the physiological status of fish at various points during capture (e.g. while in the trawl or during escape) is logistically challenging but could be coupled with attempts to quantify fish escapees/discards, with fish being sampled for physiological indicators (e.g. using a blood or tissue sample) while they are being handled or retained for the purposes of counting or monitoring.

The exact stressors and degree of stress (e.g. exhaustion, barotrauma, physical injury, hypoxia stress) that fish encounter will depend on the target species (Chopin and Arimoto, 1995; Davis, 2005; Suuronen, 2005; Broadhurst *et al.,* 2006; Benoìt *et al*., 2012), their body size (Hall and Mainprize, 2005; Halliday and Pinhorn, 2002), their interaction with the fishing gear (Cook *et al.,* 2013, 2019), the fishing procedures and gears used, (Cook *et al.,* 2019; Methling *et al*., 2017; Wilson *et al.,* 2014) and the environmental conditions (e.g. temperature, dissolved oxygen) present before, during and after capture and throughout the recovery period following escape or discard. Similarly, the most appropriate and informative biomarkers will depend on the exact stressors encountered, the time of exposure to the stressors and the potential time course for recovery (Fig. 4). Circulating levels of cortisol and blood glucose, for example, may be used as generalized indicators of stress in fish. The protracted nature of cortisol release can make it difficult to pinpoint the exact source of the stressor during a fishing event, but increased air exposure is believed to elicit a cortisol response during many forms of fishing including trawling (Methling *et al*., 2017; Beardsall *et al*., 2013). In purse seines, circulating blood cortisol levels have been observed to increase with time in the net and increased crowding (Marcalo *et al*., 2006; Tenningen *et al*., 2012). Analysis of generalized endocrine responses can be combined with more targeted biomarkers for specific insight into the type and duration of stress that fish encounter during capture by trawling during the capture process. For example, muscular concentrations of lactate, glycogen and ATP can be used to infer status after exhaustion from physical activity (Cliff and Thurman, 1984; Skomal and Mandelman, 2012), such as that which can occur while swimming away from an oncoming trawl struggling after being hooked on longlines (Roth and Rotabakk, 2012), crowding and becoming hypoxic during a purse seine or thrashing during net entanglement during active fishing (e.g. trawling or purse seining; Marcalo *et al*., 2006; Tenningen *et al*., 2012) or passive netting or trapping (Folkins *et al*., 2021). Blood plasma levels of various ions (e.g. sodium, potassium, chloride, magnesium) are also tightly linked to physiological stress in fish, and circulating levels of various enzymes (e.g. creatine kinase, lactate dehydrogenase) can indicate tissue damage, including that specific to cardiac trauma or barotrauma (Wells et al., 1986; Killen *et al.,* 2003; Suski *et al.,* 2003). Recent studies also suggest that the total amino acid composition of protein in fish ocular and muscular tissue could be used as an indicator of stress (Falco *et al.,* 2020) as they are important in cellular metabolism in response to long-term stress (Wu, 2013). Moreover, also plasma levels of catecholamines, which are immediately released upon the perception of stressors, including hypoxia, hypercapnia, exhaustive exercise and handling, could also be used to infer stress status (Mazeaud *et al.,* 1977; Randall and Perry, 1992; Skomal and Mandelman, 2012). Gene expression, as elicited via hormonal pathways in response to stressors (e.g. the negative feedback suppression by glucocorticoids of the pituitary POMC gene; Sapolsky *et al.,* 2000), can also be used to detect cellular-level responses to stressors, such as hypoxia or thermal stress, and the time courses for recovery.

## What is the relationship between physiological condition and survival?

Third, these physiological biomarkers need to be linked with the probability that fish will experience mortality or survive after exposure to a given combination of fishing procedures and conditions. This link between physiological status and survival would need to be established experimentally and, ideally, with the use of conservative, sub-lethal study endpoints (Fig. 3). Estimates of survival probability should consider not only the direct physiological disturbance and damage caused by the fishing experience itself on potential for recovery, but also any behavioural impairments that occur during recovery that may make escaped or discarded (Marcalo *et al*., 2006; Tenningen *et al*., 2012) fish more vulnerable to additional sources of mortality, including predation (Davis, 2005; Ryer *et al.,* 2004).

There are at least two main routes for calibrating physiological status with likelihood of recovery following escape after discard. The first is to sample individual fish that have actually escaped from trawls or have been captured and brought on board a boat, then monitor them during recovery (Berragan-Mendez *et al*., 2019). For example, the trawl coverings that are used to retain escapes are often left at depth, with the ‘escaped’ fish inside, to monitor their survival (Ingólfsson *et al.,* 2007; Duzbastılar *et al*., 2017). Depending on the depth involved, fish could be sampled *in situ* by divers for later analysis of blood parameters or other physiological variables, then subsequently monitored for behaviour throughout recovery. While these monitoring experiments may not give accurate depictions of the true mortality rate after escape or discard, they should still be useful for calibrating relationships between a given physiological biomarker and the probability of mortality or survival.

A second approach is to use laboratory-based simulations of the stressors encountered during the capture process to evaluate physiological status and effects on subsequent behaviour and physiology (Killen *et al.,* 2015). While it is difficult, if not impossible, to simulate all of the stressors encountered by fish during capture by trawl, this approach is useful for isolating the effects of specific stressors or potential interactions between stressors that are simultaneously measured and controlled. Both approaches (sampling of fish during actual trawling event or during experimental simulations) could also be accompanied by efforts to follow fish in the wild during recovery, without enclosures, using various innovative technologies for tracking fish movements and behaviours (Beardsall *et al.,* 2013; Gallagher *et al.,* 2017; Killen *et al.,* 2017).

Once appropriate physiological biomarkers are identified, which relate to behavioural impairments that would likely lead to mortality in the wild, stocks can be sampled during research surveys or even during commercial fishing efforts to quantify the population status with regard to these biomarkers (Fig. 3). Changes in these biomarkers could then be used to more precisely estimate effective fishing mortality from the length structure of the landed catch, by estimating the percentage of fish that can be expected to die after escape or discard. Furthermore, several authors have observed reflex impairment as a direct sign of stress, which can be easily and rapidly measured in free swimming or restrained fish responding to peripheral stimuli such as gravity, light, sound and touch (Davis, 2010). The end goal of a FEPSA would be the development of accurate biomarkers for rapid estimation of stress, damage and, hence, collateral mortality on board in the case of discards. This could be performed by physiologically calibrated visual evaluation, similar to the evaluation of reflex impairment in recreational fisheries (Davis, 2010; Methling *et al*. 2017; McLean *et al.,* 2020) or via rapid, onsite blood sampling for glucose, lactate or other factors using portable analytical devices (Gallagher *et al.,* 2010). While similar strategies are beginning to be applied in fish involved in trawl events (Methling *et al*. 2017), there is almost no knowledge of whether Mediterranean species may be suitable for such approaches and the relationships between a given visual indicator, predicted physiological state and probability of survival.

After collateral mortality has been estimated and calibrated with physiological biomarkers, efforts can be made to account for these hidden sources of mortality when estimating maximal catch rates (Jean, 1963; McLoughlin *et al.,* 1991; Alverson and Hughes, 1996; Chopin *et al.,* 1996; Harley *et al.,* 2000). More generally, accurate estimates of collateral mortality could also provide benchmarks for evaluating efforts to reduce indirect mortality occurring through stress and injury occurring during or after interactions with fishing gears. Scientists should propose realistic and feasible corrective measures suitable for use by commercial fleets with the minimal negative economic impact. As an example, there is scientific evidence (Mellon-Duval *et al*., 2010; and other references in Ragonese, 2018) of high post-release survival rates of small-sized hake (*Merluccius merluccius*) in the Mediterranean Sea. However, achieving these survival estimates are dependent on logistically challenging procedures (including a protracted recovery period and extremely delicate handling) that are unfeasible or impossible to be adopted by fishers. For example, suggestions to employ the use of specialized tanks and devices for fish recovery or transport for release (cfr. Broadhurst *et al.,* 2006) may be unrealistic for Mediterranean fishing vessels, which often have extremely limited space for additional equipment. In such cases, the best route for achieving reduced stress in captured fish may be to alter practices before fish are brought aboard a vessel, including the use grids within trawls to reduce the capture of both undersized specimens and rubbish that can cause injury, reducing trawl duration or trap ‘soak’ times (shown in Fig. 3), spraying fresh seawater on the catch during the sorting and the use of mobile tarpaulins to limit the effects of direct sunlight on drying and temperature change and, overall, returning fish to the sea as soon as possible with minimal handling and manipulation.

## Conclusions

If the status of fish populations is of great enough concern to institute the recently introduced EU directives, then the research effort required to satisfy these objectives should be of equal priority. While the EU discard ban can be viewed as a step in the right direction towards discouraging rampant bycatch and destructive discard practices, a tremendous research effort is needed to effectively implement this policy. While these challenges are faced by fisheries across Europe, the Mediterranean Sea may face the greatest challenges due to intense local fishing pressure on large numbers of species, the interactive effects of additional extrinsic stressors (e.g. thermal variation, hypoxic episodes, pollution) and complicated jurisdictional boundaries. The unnecessary removal of fish that would otherwise recover from the capture process, as is dictated by the EU regulation, is wasteful and needlessly eliminates accumulated energy from aquatic food webs and ecosystems. The granting of species exceptions to the landing obligation could avoid these consequences, but much more research is needed in this area. Furthermore, the granting of official GES by the EU is dependent upon proper evaluation of fisheries sustainability and ecosystem health; however, to date, we lack a comprehensive, integrative means of assessing these issues. We suggest the inclusion of physiological parameters and studies that could greatly assist with all these issues. This will not be a simple undertaking and will require multi-disciplinary collaborations among fisheries scientists, animal physiologists and ecologists. Therefore, we call upon scientists with expertise in these areas to recognize this effort as a priority research issue for their skillsets and for funding agencies, particularly those within the EU, to financially support the work that is needed to implement the policies that have been put in place without accumulating additional environmental damage.

## Funding

S.S.K. was supported by Natural Environment Research Council (grant NE/T008334/1) and a European Research Council Starting Grant (640004).

## Author Contributions

All authors have made substantial contributions to the conception and design of this perspective. F.F. and S.S.K. drafted the manuscript. All other authors assisted in editing, revising and providing additional intellectual content.
